# Considerations in Assessing the Evidence and Implications of Aid Displacement from the Health Sector

**DOI:** 10.1371/journal.pmed.1001364

**Published:** 2013-01-08

**Authors:** Rajaie Batniji, Eran Bendavid

**Affiliations:** Department of Medicine, Stanford University, Stanford, California, United States of America

## Abstract

Eran Bendavid and Rajaie Batniji provide a perspective on the ongoing debates about aid displacement - whether giving development aid to governments leads to reductions in their own domestic health financing.

**Linked Perspective Articles**

Batniji R, Bendavid E (2013) Considerations in Assessing the Evidence and Implications of Aid Displacement from the Health Sector. PLoS Med 10(1): e1001364. doi:10.1371/journal.pmed.1001364

Murray CJL, Dieleman JL, Lu C, Hanlon M (2013) More Data and Appropriate Statistical Methods Needed to Fully Measure the Displacement Effects of Development Assistance for Health. PLoS Med 10(1): e1001365. doi:10.1371/journal.pmed.1001365

Questions about whether, and how, giving health aid to governments leads to a reduction in health financing from domestic sources are essential to decisions about which countries receive aid, and whether aid is directed to governments. A 2010 analysis by Lu et al. made the case that for every additional US dollar of development assistance for health (DAH) from 1995 to 2006, government health expenditures from domestic sources fell by at least US$0.43 [1]. Similarly, Farag and colleagues found that each US dollar in DAH to low-income countries was associated with a US$0.14 decrease in those low-income countries' government health spending [2]. The methods used by Lu et al. have been scrutinized carefully, but their findings have led to concerns that recipient governments may waste donor resources [3]. Our previous essay in *PLOS Medicine* also questioned the analysis by Lu et al., but we have retracted it due to errors in our statistical model and reporting [4]. However, here we reaffirm the importance of understanding aid displacement and in particular, an exploration of where the money goes.

## Looking at the Issue from a Government's Perspective

Long-standing empirical evidence in the fields of public finance and development assistance suggests that the hard question is not how much money is displaced from the health sector, but rather how much money stays. Underlying the answer to this question is the view that in order to improve the well-being of its citizenry, governments spend on public goods in multiple sectors, not just health. For example, public spending on education, security, and infrastructure are all critical components of governmental efforts to improve well-being. Thus, when a government experiences or anticipates an increase in resources (as with any form of aid, including health aid), then making the best use of these resources would generally call for their distribution across multiple sectors. Development assistance to governments is an increase in income, which governments could use according to the available information about the needs and priorities of their citizens. This finding has been observed repeatedly, even in transfers within rich countries. In the United States, evidence on aid from the federal to state government, or from state to local government, suggests that recipient governments displaced 10 to 75 cents out of every dollar, regardless of what the aid was for [5]. Viewed this way, the surprising finding is not that governments redistribute resources upon receipt (or anticipated receipt) of development assistance, but rather that a substantial portion of the money remains in the intended sector.

## Where Does Health Aid End Up?

The critical issue, then, is not the extent of aid displacement, but where the displaced resources end up. The implications of displacing health aid in order to build a water chlorination plant are very different from those of buying a new vehicle fleet for the cabinet. Donors are justifiably concerned that health aid may be displaced for private gain. On the other hand, redistributing health aid may improve the lives of individuals in the recipient countries if used to address the population's needs. One empirical analysis, looking at all aid-receiving sectors, found that aid remaining in its intended sector did not work better than displaced aid, when “better” is understood as economic growth, spending in pro-poor sectors, or reductions in infant mortality [6]. Further, this study found that aid displacement was not associated with bad governance or irresponsible policies. Trying to trace how, and to which sectors, health aid may be redistributed is complicated by the multiple pathways and intermediate steps through which displaced aid may pass. However, empirical investigation may shed some light on these issues. For example, is there evidence of a rise in public spending on education in association with health aid displacement? Is there an association between aid displacement and health outcomes?

## Is Aid Displacement Universal?

The article by Lu et al. found a strong and robust association between the level of health aid given to a country, and the government's corresponding level of health expenditures from domestic sources. However, there is important heterogeneity in the association across countries. The data gathered by Lu et al. allow an estimation of country-specific aid displacement levels. We examined these data and here reaffirm that upon close examination of several country patterns, the effect does not appear uniform: some countries show evidence of displacement, while others show no change or even a rise in domestic health spending with rising health aid [4]. One explanation for the heterogeneity rests in the difficulties of obtaining accurate data on public financing of health. Donors may be justified in avoiding governments that are perceived to misuse health aid. However, prioritizing health aid based on this information would not be warranted if the underlying uncertainty in the data is responsible for the observed trends.

## Determinants of Health Aid Displacement

Another explanation for the heterogeneity may be about the design of health aid programs. An in-depth study in Rwanda, Honduras, and Thailand suggests that governments increased HIV spending from domestic sources in response to increased donor HIV funding [7]. If true, what could explain these inconsistent effects? This question could have important implications for donors if the answers have as much to do with the design of health aid as with the response of recipient governments. One possibility is that health aid may be more likely to be displaced if it is untargeted or earmarked for activities that were previously supported by domestic health spending. On the other hand, aid targeted to extend existing programs, rather than duplicate funding, may be less likely to be displaced. Project-level aid data are available to explore this hypothesis. The implication is that careful thought and design of health aid to complement recipient-country public health activities may decrease displacement even if it places the onus on donors to invest more in up-front work.

The work by Lu et al. provides us with new data to assess public financing of health, and their findings accord with previous work on health sector aid displacement. However, the finding of health aid displacement does not imply the effect is universal or mandatory. Health aid, including aid to governments, has been associated with substantial improvements in the health of individuals living in developing countries, and improving its design could increase its effectiveness even further.

## Abbreviations

DAH: development assistance for health
